# Draft genome sequence of *Deinococcus* sp. ME38 isolated from sediment of the Esmeralda Lake of the Parque Nacional Lagunas De Montebello

**DOI:** 10.1128/mra.00049-25

**Published:** 2025-11-05

**Authors:** Alejandra Osorio-González, Betsy Anaid Peña-Ocaña, Nancy Abril Martínez-López, José Humberto Castañón-González, Roberto Marín-Paredes, Ricardo Jasso-Chávez, Víctor Manuel Ruíz-Valdiviezo

**Affiliations:** 1 Laboratorio de Biología Molecular, Tecnológico Nacional de México/Instituto Tecnológico de Tuxtla Gutiérrez 183420, Tuxtla Gutiérrez, Chiapas, México; 2 Departamento de Bioquímica, Instituto Nacional de Cardiología, Mexico City, México; 3 Laboratorio de Microbiómica, Escuela Nacional de Estudios Superiores Unidad Morelia, UNAM 373249 , Morelia, Michoacán, México; Wellesley College, Wellesley, Massachusetts, USA

**Keywords:** bioaccumulation, radioactive bacteria, freshwater lake

## Abstract

We report the complete genome of *Deinococcus* sp. strain ME38, which was isolated from sediment of Lake the Parque Nacional Lagunas de Montebello. This genome consists of 4,397,810 bp and 4,474 coding sequences, with 69.3% G + C content. Whole-genome sequencing was performed to explore the bioremediation potential in aquatic ecosystems.

## ANNOUNCEMENT

Freshwater ecosystems, such as lakes, can host a wide diversity of bacteria, including extremophiles, which thrive in extreme microhabitats with minimal energy cost for growth (1). This phenomenon is explained by the Baas Becking hypothesis, which suggests that microorganisms are found everywhere, but the environment selects their presence (2). According to this hypothesis, radioresistant species of the *Deinococcus* genus have been reported to thrive in aquatic ecosystems (3). These gram-positive bacteria can mitigate damage caused by various stresses (ionizing radiation, UV radiation, oxidation, deactivation, and mitomycin C) (4) and have been noted for their biosorption (5, 6) and bioaccumulation capabilities of heavy metals (7, 8). While radioresistance has been described as a general trait of some *Deinococcus* members, no direct evidence is currently available to confirm this characteristic in the isolate from Lake Montebello.

*Deinococcus* sp. ME38 strain was isolated from sediment from the Esmeralda Lake of El Parque Nacional Lagunas de Montebello, Chiapas, Mexico. The sediment was collected at a distance of 1 m from the shore of the lake (16°7'6''N, 91°43'42''W). The sediment collection took place on 18 August 2017. For bacterial cultures, 1 g of sediment was suspended in 10 mL of sterile distilled water, and serial dilutions were made, with the 10^−6^ dilution spread on plates. The medium used was BBR (10 g/L peptone, 5 g/L yeast extract, 10 g/L NaCl, 0.5 g/L K_2_HPO_4_, 0.2 g/L MgSO_4_·7H_2_O; pH adjusted to 7.2), and incubation was at 20°C for 10 days (9). Isolates were selected based on colony morphology, and although ME38 belongs to the *Deinococcus* genus, its radioresistance has not yet been experimentally validated. Strain ME38 was isolated on TGY medium with 15 g agar using the cross-streak method and incubated at 25°C for 72 h (10). The ME38 isolate was taxonomically identified as belonging to the *Deinococcus* genus by 16S rRNA gene sequencing, followed by BLAST analysis against the NCBI database, which showed >99% similarity with *Deinococcus* spp. This identification was further supported by average nucleotide identity analysis, which confirmed its placement within the *Deinococcus* genus.

**Fig 1:**
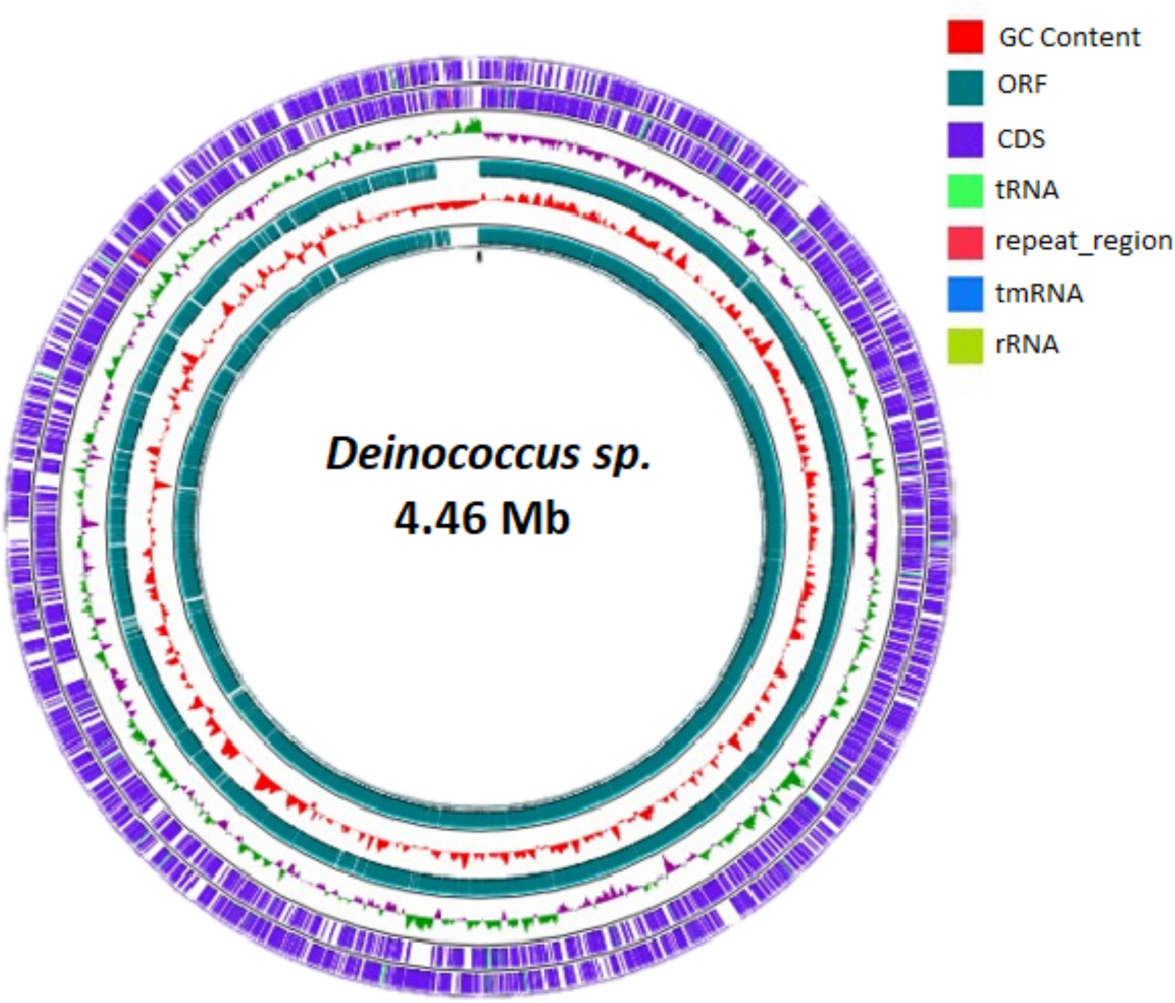
Circular map of the complete genome of *Deinococcus* sp. The map was made with GC view server.

Total genomic DNA from strain ME38 was extracted using the commercial ZR Fungal/Bacterial DNA MiniPrep kit (Zymo Research), and the concentration and elimination of impurities were performed with the DNA Clean & Concentrator-5 kit (Zymo Research). Subsequently, genomic DNA libraries were then created with the Illumina NextSeq platform at Integrated Microbiome Resource (IMR) (Halifax, Canada) following the manufacturer’s protocol without modifications. These libraries were then subjected to sequencing on an Illumina MiSeq system, thus generating 301 bp paired-end reads. The quality of the reads was assessed with FastQC v.1.0.1, while low-quality reads and sequence adapters were removed with Trimmomatic v0.36. Genome assembly was performed with SPAdes from the KBase Predictive Biology platform (11). Default parameters were used for all tools unless otherwise specified. We obtained a genome size of 4,397,810 bp, with a G + C content of 69.3%. A total of 201 contigs were obtained that had N_50_ values of 98,263. Genomic map construction was done with the GCview in Java software v.11 (Fig. 1). The contigs were annotated using the Rapid Annotations Subsystems Technology (RAST) v2.0 Toolkit. A total of 4,474 coding genes, 260 subsystems, and 57 RNA genes were identified.

Genome annotation for gene localization and genome functions was performed with RAST—The Seed Server in conjunction with PROKKA v1.15.5 (11), and the NCBI Prokaryotic Genome Annotation Pipeline (PGAP, version 6.9) was added. After assembly (SPAdes), redundant end sequences were identified, followed by the BLAST comparison. Then the overlap is identified and trimmed to avoid sequence duplication. A visual diagram of the circular assembly of the *Deinococcus* genome was created using the GC view server. We found two genes involved in the synthesis of cysteine and polyphosphate; these metabolites have the ability to chelate and store metals, which are available in the cytoplasm (12, 13); two genes related to the production of cadmium and copper resistance proteins; three genes involved in the production of lead, cadmium, zinc, and mercury transporting ATPases; and finally, three genes for copper translocating P-type ATPases. This system of genes helps to expel cadmium and copper from entering the cell (14, 15).

## Data Availability

This whole genome shotgun project has been deposited at GenBank under the accession number JBMEVT000000000. The sequenced genome is publicly available under SRA, BioProject, and BioSample numbers, which are SRR32808879, PRJNA1223931, and SAMN46830730, respectively. This genome is publicly available.
